# Reverse hybrid jig separation efficiency estimation of floating plastics using apparent specific gravity and concentration criterion

**DOI:** 10.3389/fchem.2022.1014441

**Published:** 2022-09-28

**Authors:** Theerayut Phengsaart, Chaiwat Manositchaikul, Palot Srichonphaisarn, Onchanok Juntarasakul, Kreangkrai Maneeintr, Sanghee Jeon, Ilhwan Park, Carlito Baltazar Tabelin, Naoki Hiroyoshi, Mayumi Ito

**Affiliations:** ^1^ Department of Mining and Petroleum Engineering, Faculty of Engineering, Chulalongkorn University, Bangkok, Thailand; ^2^ Division of Sustainable Resources Engineering, Faculty of Engineering, Hokkaido University, Sapporo, Japan; ^3^ Department of Materials and Resources Engineering Technology, College of Engineering and Technology, Mindanao State University-Iligan Institute of Technology, Iligan City, Philippines

**Keywords:** recycling, plastic, polyolefin, gravity separation, bubble attachment

## Abstract

We developed a technique called the reverse hybrid jig, an advanced physical separation technique that combines the principles of jig and flotation to separate floating plastics. This technique is a promising green technology that is more economical and environmentally friendly compared with the conventional flotation. Although the applicability of this technique to separate PP/PE have been reported, the index to illustrate the possibility of separation for the reverse hybrid jig is still not available. In this study, a reverse apparent concentration criterion (*CC*_*RA*_) is proposed to estimate reverse hybrid jig separation efficiency. This modified concentration criterion can be calculated using the specific gravity (*SG*) of particle with attached bubbles called the apparent specific gravity (*SG*_*A*_). To determine the volume of attached bubbles on plastic surfaces under water pulsation, a laser-assisted apparatus was used under various conditions, including plastic type, air flow rate, dosage, and type of wetting agent. The results of attached bubble volume measurements were used to calculate the *SG*_*A*_ and *CC*_*RA*_. The estimated values were then compared with the results of reverse hybrid jig separation. It was found that higher *CC*_*RA*_ resulted in better separation efficiency. In addition, an empirical linear equation for estimating the reverse hybrid jig separation efficiency is proposed.

## 1 Introduction

Management of municipal solid wastes (MSW) is a big global challenge particularly because of the increasing amount of their associated plastic waste fraction (Geyer et al., 2017). During the Coronavirus disease (COVID-19) pandemic, the situation with plastic wastes worsened as consumption of single-use plastics in households skyrocketed. Various types of single-used plastics (e.g., surgical mask, plastic glove, and packaging from food delivery) are not treated properly and often mixed with MSW. Prata et al. (2020), for example, estimated that the monthly use of personal protective equipment (PPE) worldwide was approximately 129 billion face masks and 65 billion gloves. Furthermore, lockdowns in many countries around the world due to the pandemic led to the growth of e-commerce and food delivery services that increased plastic packaging waste generation by 53% in 2021 (Filho et al., 2021).

Almost half of plastics produced worldwide (46%) and more than half of plastic used in the global packaging industry (69.5%) is made up of polyolefin (Rabnawaz et al., 2017). Polyolefin—floating plastics like polypropylene (PP) and polyethylene (PE)—is the most widely produced and consumed type of thermoplastics due to its low cost, light weight, easy processability, and good recyclability (Gopanna et al., 2019). Moreover, PP is one of the most important plastics used to fabricate medical face masks (Irez et al., 2022).

Resources recovery, the concept of waste reduction by extracting valuable materials from waste streams, is a promising strategy for the management of plastic wastes in MSW. Recovered plastic wastes can be recycled to generate new plastic materials (i.e., resources recycling) and/or used as supplementary fuel for cement production, pyrometallurgical operations and electricity generation (i.e., energy recovery) (Tabelin et al., 2021). Compared with landfilling, incineration, and energy recovery, resources recycling is a more sustainable approach because plastic materials are recirculated back into the economy (Demirbas, 2011). Therefore, resources recycling is an important way of minimizing the amounts of plastic wastes disposed of in landfills or burned in incinerators. It means that recycling of mixed plastic wastes is critical to limit the negative environmental impacts of plastics as well as to conserve finite natural resources. It is also included in the United Nation Sustainable Development Goals (UN-SDGs): Goal 12 “Responsible production and consumption” (United Nations, 2015). To effectively recycle plastics, separation of various plastic types in mixed-plastic wastes is essential. Sink-float separation in water is commonly used to separate polyolefins from other mixed plastics because they have lower densities than water causing them to float while other types of plastics sink with their heavier density. The sink-float separation of various polyolefins like PP and PE, however, is expensive because it requires the use of water-ethanol mixtures as medium (Mumbach et al., 2019).

**FIGURE 1:**
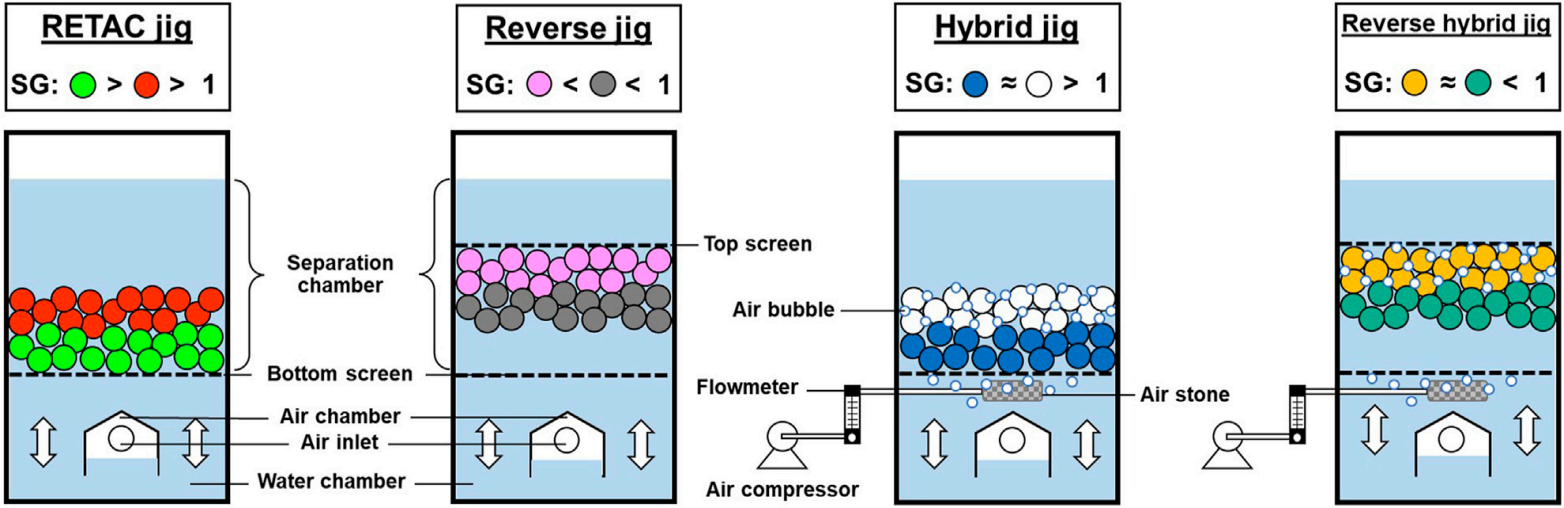
Schematic illustrations of advanced jigs.

An alternative to sink-float separation for the separation of mixed-plastics is the use of jig, a gravity concentration technique that uses vertical expansion and contraction of a bed of particles by a pulse of fluid to separate the particle based on difference of specific gravity (*SG*) and settling velocity (*v*). It is one of the oldest mineral processing methods and still used due to its simple operation, low cost, and high efficiency. Among the many types of jigs, four advanced jig separation technology—RETAC jig, reverse jig, hybrid jig, and reverse hybrid jig (Figure 1)—were developed in the Laboratory of Mineral Processing and Resources Recycling, Hokkaido University, Japan for plastic-plastic separation. First, the RETAC jig, modified from coal cleaning TACUB (Tsunekawa Air Chamber Under Bed) jig (commercially called as BATAC jig) (Hori et al., 2009a; Tsunekawa et al., 2012; Phengsaart et al., 2020, 2021), was successfully applied to recover plastics and other valuable materials from copy machines (Tsunekawa et al., 2005), automobile shredded residues (ASR) (Kuwayama et al., 2014), small home appliances (Phengsaart et al., 2018), mobile phones (Jeon et al., 2019) in both laboratory- and pilot-scales. Second, the reverse jig, which is the RETAC jig with top screen, was developed to separate plastics lighter than water (e.g., PP and high-density polyethylene (HDPE) from packaging containers) by their difference in levitating velocity (Ito et al., 2010). Third, the hybrid jig was developed to separate plastics with similar *SG*s but different surface wettabilities by combining density- and surface-based separation concepts. In this type of jig, an aeration tube is installed under the screen to generate air bubbles introduced into the jig separation chamber. Separation occurs when the *SG* of one type of plastic is reduced and changed into apparent specific gravity (*SG*_*A*_) due to the attachment of bubbles. This causes stratification during water pulsation, so the lower *SG*_*A*_ (more hydrophobic) plastic is recovered on top while that with higher *SG*_*A*_ (more hydrophilic) is recovered at the bottom. The hybrid jig effectively separated various types of mixed-plastics like polyethylene terephthalate (PET)/polyvinyl chloride (PVC) (Hori et al., 2009b; Ito et al., 2019a, 2020), polypropylene with glass fiber (PPGF)/high impact polystyrene (HIPS) (Ito et al., 2019b, 2020), and PVC/polyamide (nylon-66 or PA) (Ito et al., 2019b). Finally, the reverse hybrid jig—a combination of reverse and hybrid jigs—was developed to separate floating plastics having similar *SG*s like PE/cross-linked polyethylene (XLPE) mixtures (Ito et al., 2021).

For separation to occur, less selectivity of bubble attachment is required for hybrid and reverse hybrid jigs compared with flotation. Very high bubble attachment selective is crucial in flotation because bubbles are used to separate and carry hydrophobic particles to froth products while hydrophilic particles are left in suspension. Because of this, the high consumption of chemical reagents (e.g., wetting agents and frothers) are required for high selectivity and efficiency (Aikawa et al., 2020; Hornn et al., 2020, 2021). In contrast, only few bubbles with low selectivity are needed for effective separation in hybrid and reverse hybrid jigs because their main role is only to change the *SG* of more hydrophobic plastics. Meanwhile, water pulsations are introduced to stratify the particles into different layers in the separation chamber, so little consumption of chemical reagents is required (Ito et al., 2019b). Therefore, advanced jigs are both green and sustainable technologies.

To obtain high quality recycled plastics with similar physical/chemical properties of virgin resins, high separation efficiency is required. In the previous studies of the authors about advanced jig separation efficiency, the concentration criterion (*CC*)—an index illustrates the possibility of gravity separation—was modified to estimate the separation efficiency of the RETAC jig, reverse jig, and hybrid jig. However, the estimation of *CC* for floating plastics using the reverse hybrid jig separation is still lacking in the literature.

In this study, a laser-assisted measurement apparatus was used to measure attached-bubble volume on plastics during water pulsation and determine the *SG*_*A*_ of plastics under various conditions (i.e., plastic type, air flow rate, dosage and type wetting agents). Finally, a new index based on *CC* is proposed to estimate the separation efficiency of reverse hybrid jig separation.

## 2 Concentration criteria for advanced jig separation

The concentration criterion (*CC*) is an index used to determine the possibility of separation by gravity concentrators like conventional jigs. The higher the value of *CC*, the easier it is to separate materials in the sample (Wills and Finch, 2015; Gupta and Yan, 2016). It is calculated using the following equation (Wills and Finch, 2015; Gupta and Yan, 2016):

$$CC=\frac{\rho_{H}-\rho_{F}}{\rho_{L}-\rho_{F}}$$ (1)

where *ρ*_*H*_ and *ρ*_*L*_ are the mass density of heavy (*H*) and light (*L*) materials, respectively while *ρ*_*F*_ is the mass density of fluid medium used for separation.

In terms of *SG*, Eq. 1 can be rewritten as

$$CC=\frac{{SG}_{H}-{SG}_{F}}{{SG}_{L}-{SG}_{F}}$$ (2)

where *SG*_*H*_ and *SG*_*L*_ are the specific gravities of *H* and *L* materials, respectively while SG_F_ is the specific gravity of fluid medium.

In gravity separation, particles with different densities are separated by the difference of their settling velocities (v) in fluid usually water. The settling of a particle in water is strongly influenced by three forces—gravity force (*F*_*G*_; Eq. 3), buoyancy force (*F*_*B*_; Eq. 4) and drag force (*F*_*D*_; Eq. 5)—while its *v* is determined by the balance between these three forces (Eq. 6).

$$F_{G}=mg$$ (3)

$$F_{B}=\frac{m}{\rho_{P}}\rho_{F}g$$ (4)

$$F_{D}=C_{D}A\frac{\rho_{F}v^{2}}{2}$$ (5)

$$m\frac{dv}{dt}=mg-\frac{m}{\rho_{P}}\rho_{F}g-C_{D}\left(\frac{\rho_{F}v^{2}}{2}\right)$$ (6)

where *m* is the mass of the particle [kg], *v* is the velocity of particle [m/s], *t* is time [s], *g* is gravitational acceleration [m/s^2^], *ρ*_*P*_ is density of particle [kg/m^3^], *ρ*_*F*_ is density of fluid [kg/m^3^], *C*_*D*_ is drag coefficient, and *A* is the projection area of particle [m^2^].

$$m=\rho_{P}V$$ (7)

where *V* = volume of particle [m^3^].

When a particle reaches terminal velocity (*v*_*∞*_), its acceleration (*dv/dt*) becomes zero and Eqs 6, 7 can be simplified to the following equation:

$$0=\left(\rho_{P}-\rho_{F}\right)gV-C_{D}A\left(\frac{\rho_{F}v_{\infty }^{2}}{2}\right)$$ (8)

If the particle is a sphere (^*^), its projection area (*A* ^*^) and volume (*V* ^*^) are given by Eqs 9, 10.

$$A^{*}=\frac{\pi D^{2}}{4}$$ (9)

$$V^{*}=\frac{\pi D^{3}}{6}$$ (10)

The drag coefficient (*C*_*D*_) in Eqs 5, 6 could also be expressed as a function of the Reynolds number (*Re*_*p*_) assuming a laminar flow regime during particle settling (*Re*_*p*_ < 1) (Eqs 11, 12).

$${Re}_{p}=\frac{vD\rho_{f}}{\mu }$$ (11)

$$C_{D}=\frac{24}{{Re}_{p}}$$ (12)

where *µ* is the viscosity of fluid [Pa,s].

In a laminar or Stokes’ flow regime, the terminal velocity of a sphere (*v*_∞_^*^) could be calculated and is given as Eq. 13 by substituting Eqs 9–12 to Eq. 8.

$$v_{\infty }^{*}=\frac{\left(\rho_{P}-\rho_{F}\right)gD^{2}}{18\mu }$$ (13)

Using Stokes’ law (Eq. 13), *CC* (Eq. 1) can be expressed by the ratio of *v*_∞_ of heavy and light particles.

$$CC=\frac{v_{\infty H}^{*}}{v_{\infty L}^{*}}$$ (14)

where *v*_∞_^*^_H_ and *v*_∞_^*^_L_ are the terminal velocities of heavy and light materials with spherical shape, respectively.

The equations of conventional *CC* (Eqs 1, 2 and Eq. 14), however, are not applicable for non-spherical particles. To modify the *CC* and capture the effects of particle shape, a dynamic shape factor called shape settling factor (*SSF*) was introduced (Eq. 15) (Gupta and Yan, 2016).

$$SSF=\frac{v_{\infty }}{v_{\infty }^{*}}$$ (15)

The *SSF* is defined as the ratio of the terminal velocity in static water of a non-spherical particle (*v*_*∞*_) and the terminal velocity in static water of a spherical particle having the same *D* (*v*_∞_^*^). Using *SSF* (Eq. 15), the modified concentration criterion for non-spherical particles (*CC*_*S*_) were obtained as follows (Pita and Castilho, 2016):

$${CC}_{S}=\frac{\rho_{H}-\rho_{F}}{\rho_{L}-\rho_{F}}\times \frac{{SSF}_{H}}{{SSF}_{L}}$$ (16)

where *SSF*_*H*_ and *SSF*_*L*_ are the shape settling factor of heavy and light materials, respectively.

Then,

$${CC}_{S}=\frac{\rho_{H}-\rho_{F}}{\rho_{L}-\rho_{F}}\times \frac{\frac{v_{\infty H}}{v_{\infty H}^{*}}}{\frac{v_{\infty L}}{v_{\infty L}^{*}}}$$ (17)

where *v*_*∞H*_ is the terminal velocity in static water of a heavy, non-spherical particle, *v*_∞L_ is the terminal velocity in static water of a light, non-spherical particle, v_∞_^*^_H_ is the terminal velocity in static water of heavy, spherical particle having the same *D* as the heavy, non-spherical particle, and *v*_∞_^*^_L_ is the terminal velocity in static water of a light, spherical particle having same *D* as a light, non-spherical particle.

Finally,

$${CC}_{S}=\frac{v_{\infty H}}{v_{\infty L}}$$ (18)

However, *CC* and *CC*_*S*_ are suitable for estimate the separation efficiency of sinking plastics using conventional and RETAC jigs. To estimate the results of reverse jig separation that separate the particles based on levitation velocity difference, Eqs 1, 2 were modified into reverse concentration criterion (*CC*_*R*_) as shown in Eq. 19 (Ito et al., 2010).

$${CC}_{R}=\frac{{SG}_{L}-{SG}_{F}}{{SG}_{H}-{SG}_{F}}$$ (19)

Similarly, for hybrid jig separation, since the apparent specific gravity (*SG*_*A*_)—*SG* changed by bubble attachment—affects the separation efficiency more than the inherent *SG*s of plastics therefore, *SG*_*A*_ (Eq. 20) calculated from the inherent specific gravity of materials (*SG*_*P*_) and volume of attached bubbles on particles during water pulsation (*V*_0_^*^) need to be considered (Ito et al., 2020).

$${SG}_{A}=\frac{{SG}_{P}}{1+V_{0}^{*}}$$ (20)

From Eqs 2, 20, the apparent concentration criterion (*CC*_*A*_) —modified *CC* based on *SG*_*A*_—was proposed as Eq. 21 to determine the efficiency of hybrid jig separation (Ito et al., 2020).

$${CC}_{A}=\frac{{SG}_{AH}-{SG}_{F}}{{SG}_{AL}-{SG}_{F}}$$ (21)

where *SG*_*AH*_ and *SG*_*AL*_ are the *SG*_*A*_ of *H* and *L* materials, respectively.

These *CC*, *CC*_*S*_, *CC*_*R*_, and *CC*_*A*_ have been successfully applied to estimate the separation efficiency of various jig separation of plastics (Ito et al., 2010, 2020; Pita and Castilho, 2016). However, an index to illustrate the possibility of separation for reverse hybrid jig is still not available. In this study, reverse apparent concentration criterion (*CC*_*RA*_)—modified *CC* combining the principles of reverse jig and hybrid jig (Eqs 19, 21)—was proposed to estimate reverse hybrid jig separation efficiency (Eq. 22) The experiments to confirm this proposed equation were carried out as explained in the next section.

$${CC}_{RA}=\frac{{SG}_{AL}-{SG}_{F}}{{SG}_{AH}-{SG}_{F}}$$ (22)

## 3 Materials and methods

### 3.1 Samples

The samples used in this study are virgin plastic pellets about 3.5–4.0 mm in size, spherically shaped and without additives. Two types of polyolefins, PP (PP1100NK, POLIMAXX, IRPC Public Co., Ltd., Thailand) with *SG* of 0.90 and PE, which is further subdivided into low-density polyethylene (LDPE; LDJJ4324, POLENE, TPI Polene Public Co. Ltd., Thailand) with *SG* of 0.92 and high-density polyethylene (HDPE; HD2308J, InnoPlus, PTT Global Chemical Public Co., Ltd., Thailand) with *SG* of 0.96.

### 3.2 Reagents

Four types of wetting agents were used: 1) docusate sodium salt (Aerosol OT; AOT), 2) calcium lignosulfonate (CaLS), 3) sodium lignosulfonate (NaLS), and 4) tannic acid (TA). Methyl isobutyl carbinol (MIBC), a reagent widely utilized in flotation as a frother, was used to stabilize bubbles in solution. All chemicals used in the experiments are reagent grade obtained from Sigma-Aldrich Co., United States .

### 3.3 Measurements of attached-bubble volume

**FIGURE 2:**
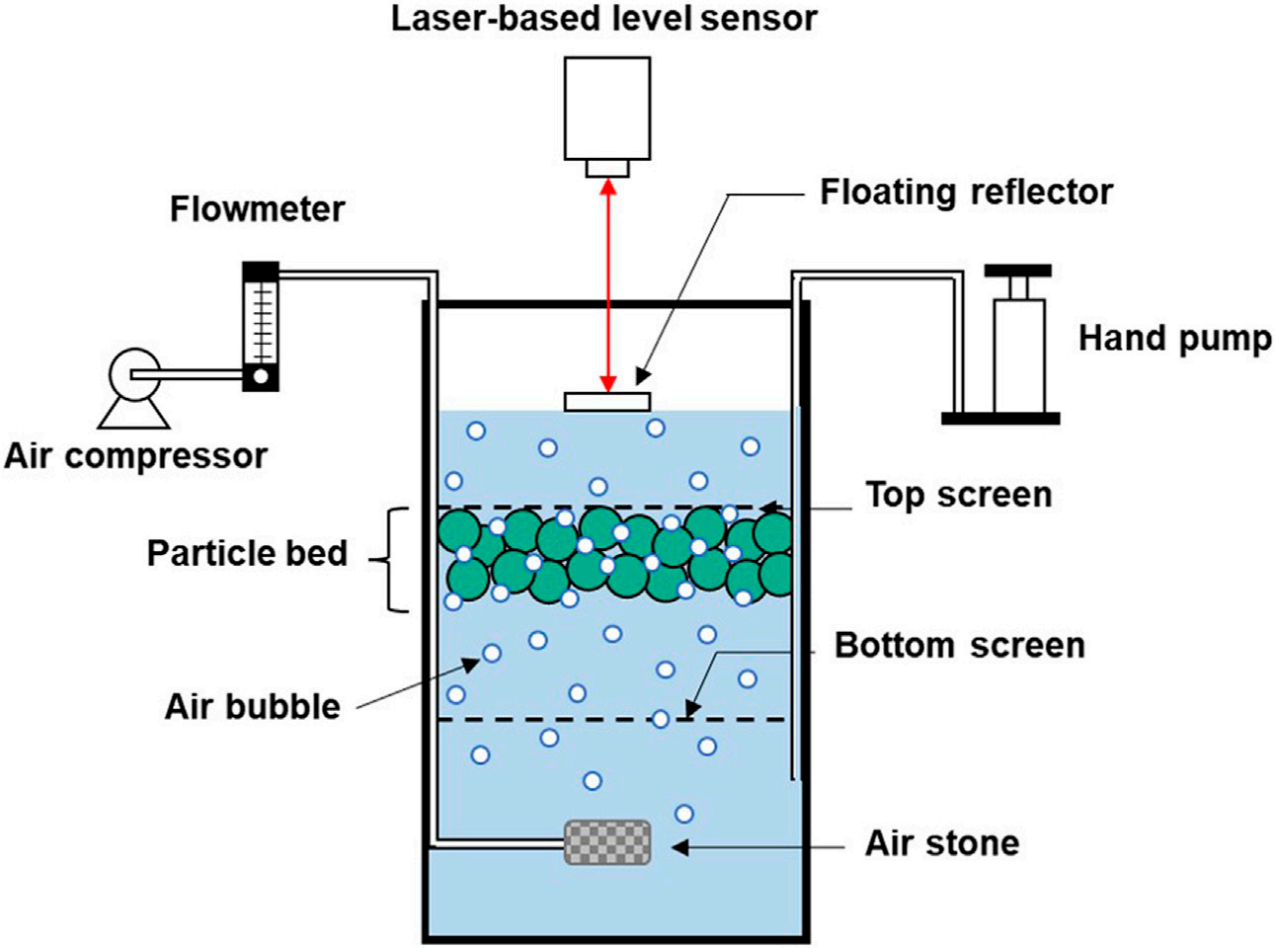
A schematic diagram of the laser-assisted measurement setup for the determination of attached-bubble volume.

Because *SG*_*A*_ (Eq. 20) is an important factor to estimate the reverse hybrid jig separation efficiency, determination of attached-bubble volume on plastic surfaces during water pulsation (*V*_0_^*^) were carried out under various conditions using the apparatus shown in Figure 2 (Ito et al., 2020, 2021).

In these tests, a reverse hybrid jig with a separation chamber 60 mm long, 60 mm wide, and 150 mm high were used. A hand-pump connected to the air chamber was used for water pulsation while an air pump introduces air into the jig through a tube connected to porous rock for creating air bubbles under the separation chamber. Air bubbles are introduced under the particle bed, and when bubbles attached to particles, an equivalent water level rise is recorded. This water level rise is accurately measured and recorded by a laser-based level sensor (HG-C1050, Panasonic Corporation, Japan) with a floating reflector on top of the water surface. The attached-bubble volume can then be calculated from changes in water level inside the separation chamber before and after bubble introduction (Ito et al., 2020, 2021).

**Supplementary Figure S1 **shows the procedure of this measurement in which 50 g of a single-type plastic sample (i.e., PP, LDPE, or HDPE) was fed into a separate chamber then, 1.2 L of water containing 20 ppm of MIBC were added. The experiments were carried out with wetting agents (i.e., AOT, CaLS, NaLS, or TA) at concentrations of 0, 50, and 100 ppm. The water pulsation was controlled by hand-pump at a displacement of 20 mm and frequency equal to 30 cycles/min with air bubbles generated using an air pump at flow rates of 0.5, 1, and 1.5 L/min for 3 min. The measurement was carried out by following the procedure in the published paper of Ito et al. (2020, 2021). After finished the aeration with water pulsation, the change in water height were recorded using a laser-based level sensor. Then, four more water pulsation were introduced to remove air bubbles that were trapped in the void between plastic pallets and the height of the water surface of each pulsation were also recorded. These data were used to determine the *V*_0_^*^ (Ito et al., 2020).

### 3.4 Reverse hybrid jig separation experiments

The reverse hybrid jig separation experiments were carried out using the same reverse hybrid jig used in the attached-bubble volume measurements. 60 g of PP/LDPE, and PP/LDPE mixtures with 1:1 mixing ratio (by weight) were used for the experiments. All conditions were the same as that of the attached-bubble volume measurements. Experiments were carried out under two different methods below (Supplementary Figure S2) (Ito et al., 2021):

#### 3.4.1 Reverse hybrid jig separation experiments with continuous bubble generation

In these tests, air bubbles were introduced for 5 min as a kind of “conditioning” process. This was followed by water pulsation for 3 min with continuous bubble generation bubble. After the separation, products were collected into three layers from the top. The products in each layer were then separated by sink-float separation using 50 wt% ethanol (prepared using 99.9% C_2_H_6_O, AR grade, Qchemical Co. Ltd., Thailand) solution (SG: ∼0.91) as medium to determine the purity of each layer.

#### 3.4.2 Reverse hybrid jig separation experiments without bubble generation during pulsation

Similar to the tests in Section 3.4.1, air bubbles were introduced for 5 min, but after conditioning, air introduction was stopped and water pulsation was introduced for 3 min. For the determination of purity of products in each layer, the same sink-float separation method was used as outlined previously.

## 4 Results and discussion

### 4.1 Effects of air flow rate and wetting agents on the apparent specific gravity.

**FIGURE 3:**
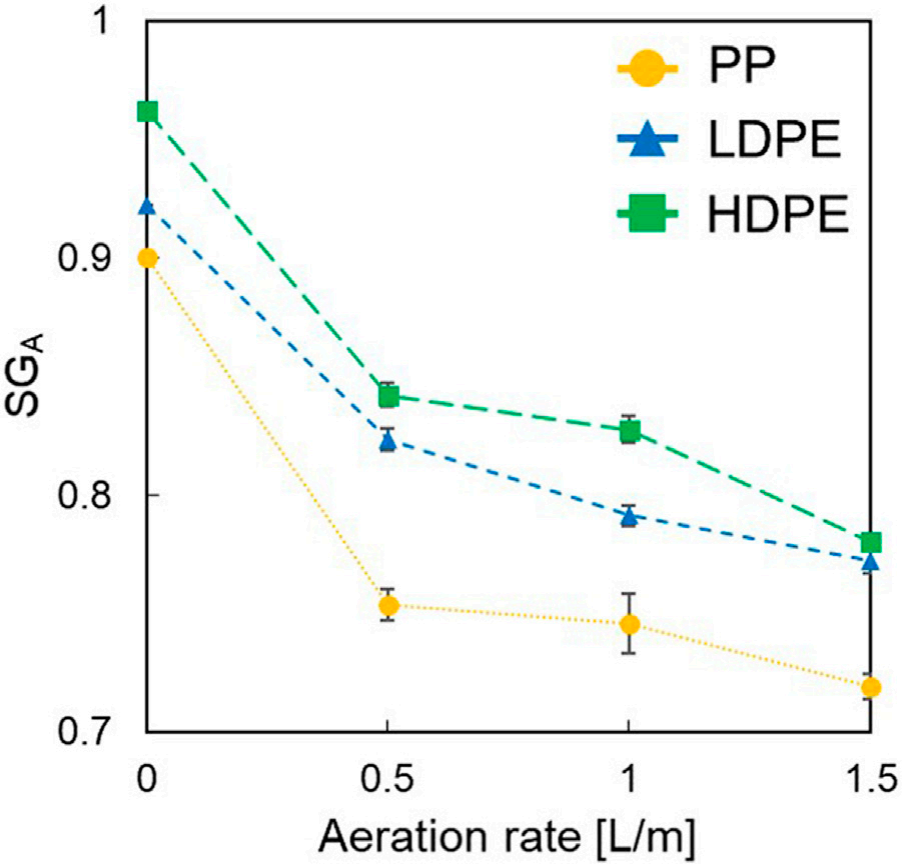
Apparent specific gravities of PP, LDPE, and HDPE at different air flow rates (i.e., 0, 0.5, 1.0, and 1.5 L/min) in water (i.e., without wetting agent).

Figure 3 shows the *SG*_*A*_ of plastics measured using the laser-assisted measurement apparatus without wetting agent at air flow rates of 0, 0.5, 1.0, and 1.5 L/min. HDPE had the highest *SG*_*A*_ regardless of the air flow rate and was followed by LDPE and PP. It was also found that the *SG*_*A*_ of all types of plastics decreased with increasing air flow rate, which could be attributed to the higher volume of bubbles attached to plastic surfaces (Eq. 20).

After determining the values of *SG*_*A*_, these were used to calculate *CC*_*RA*_ using Eq. 22, and the results are presented in Table 1. The plastic mixtures of PP/HDPE showed higher *CC*_*RA*_ compared with that of PP/LDPE because of larger differences in *SG*_*A*_ of the former than the latter. It is also interesting to note was also found that the *CC*_*RA*_ were higher when lower air flow rate was used. These results indicate that the conditions with lower air flow rate should achieve higher separation efficiency if the proposed equation (Eq. 22) is correct as well as the separation of PP/HDPE should be better than that of PP/LDPE.

**TABLE 1:** Reverse apparent concentration criterion (*CC*_*RA*_) of each plastic mixtures (i.e., PP/LDPE and PP/HDPE) and sharpness index (*SI*) of reverse hybrid jig separation experiments without bubble generation during pulsation at air flow rate (i.e., 0.5, 1.0, and 1.5 L/min) and wetting agent of 0 ppm.

Plastic mixtures	Air flow rate [L/min]	*CC*_*RA*_	*SI*
PP/LDPE	0.5	1.39	0.95
	1.0	1.22	0.96
	1.5	1.23	0.88
PP/HDPE	0.5	1.56	0.82
	1.0	1.47	0.94
	1.5	1.28	0.77

**FIGURE 4:**
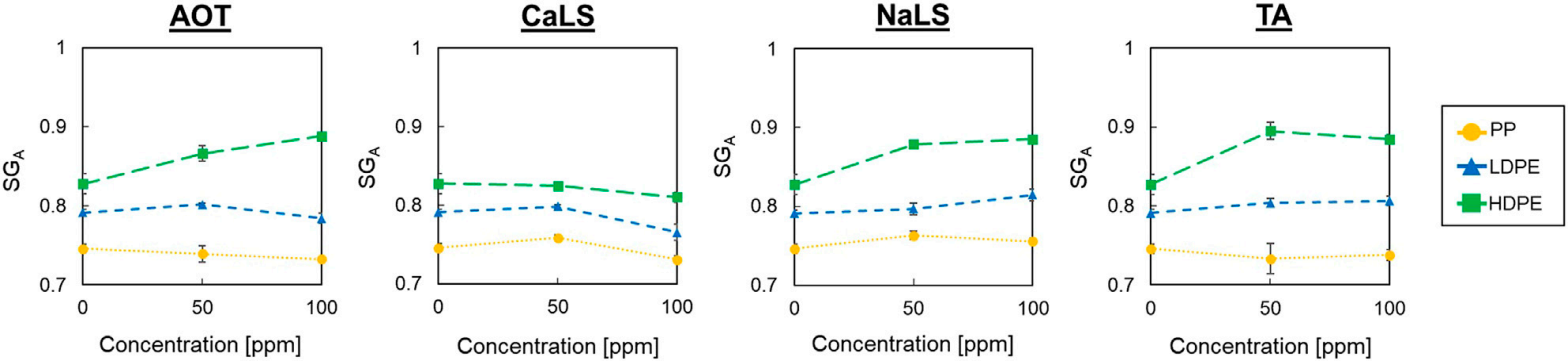
Apparent specific gravities of PP, LDPE, and HDPE at air flow rate of 1.0 L/min with AOT, CaLS, NaLS, and TA at concentrations of 0, 50, and 100 ppm.

Figure 4 shows the *SG*_*A*_ of PP, LDPE, and HDPE at air flow rate of 1.0 L/min in the solution with wetting agent (i.e., AOT, CaLS, NaLS, and TA) of 0, 50, and 100 ppm. HDPE shows the highest *SG*_*A*_ in all conditions followed by LDPE and PP, a trend similar to the *SG*_*A*_ results in water (Figure 3). It was also found that *SG*_*A*_ of HDPE drastically changed with the concentration and type of wetting agents but those of LDPE and PP changed only slightly. To explore these discrepancies more, *CC*_*RA*_ were calculated from *SG*_*A*_ of each condition as shown in Table 2. The PP/HDPE mixtures always had higher *CC*_*RA*_ compared with the PP/LDPE mixtures under the same conditions due to their higher *SG*_*A*_ difference. Also, most conditions with wetting agents had higher *CC*_*RA*_ than without wetting agents when the same air flow rate (i.e., 1.0 L/min) was used, indicating that the addition of wetting agents could improve the reverse hybrid jig separation efficiency if this proposed index (Eq. 22) is correct.

**TABLE 2:** Reverse apparent concentration criterion (*CC*_*RA*_) of each plastic mixtures (i.e., PP/LDPE and PP/HDPE) and sharpness index (*SI*) of reverse hybrid jig separation experiments without bubble generation during pulsation at different air flow rate of 1.0 L/min and wetting agents (i.e., AOT, CaLS, NaLS, and TA) of 50 and 100 ppm.

Separation test	Wetting agents		*CC*_*RA*_	SI
	Types	Concentrations [ppm]		
PP/LDPE	AOT	50	1.31	0.93
		100	1.24	0.78
	CaLS	50	1.20	1.00
		100	1.15	1.00
	NaLS	50	1.17	0.95
		100	1.32	0.97
	TA	50	1.36	1.00
		100	1.35	0.87
PP/HDPE	AOT	50	1.95	0.52
		100	2.40	0.49
	CaLS	50	1.38	1.00
		100	1.42	0.96
	NaLS	50	1.96	0.47
		100	2.13	0.54
	TA	50	2.54	0.50
		100	2.27	0.50

The *SG*_*A*_ of HDPE increased with the addition of AOT with the highest *SG*_*A*_ difference and *CC*_*RA*_ value between PP and HDPE obtained at 100 ppm. This means that 100 ppm AOT could achieve better separation efficiency than pure water or lower AOT concentrations. For NaLS and TA, the *SG*_*A*_ of HDPE only increased until 50 ppm. After this dosage, the *SG*_*A*_ like that of AOT remained constant. Meanwhile, contrast, CaLS had negligible effects on the *SG*_*A*_ of HDPE. Also, because this agent has very low effect influence on the apparent density of the three plastics. It can be said that it does not change the hydrophobicity of the three plastics. These results indicate that AOT, NaLS, and TA are capable of selectively modifying the hydrophobic surface of HDPE to become more hydrophilic. It follows that surface modification using wetting agents like AOT, NaLS, and TA could enhance the separation of PP/HDPE since *CC*_*RA*_ could be increased.

In the case of PP/LDPE mixtures, the results are still unclear and difficult to interpret due to the unnoticeable changes of *SG*_*A*_. Based on the *CC* guide for gravity separation of Gupta and Yan (2016), PP/LDPE separation might not because particles with 3.5–4.0 mm size requires *CC* to be larger than 1.7. To confirm these hypothesis, the reverse hybrid jig separation experiments with the condition above were carried out.

### 4.2 Effects of air flow rate and wetting agents on reverse hybrid jig separation

**FIGURE 5:**
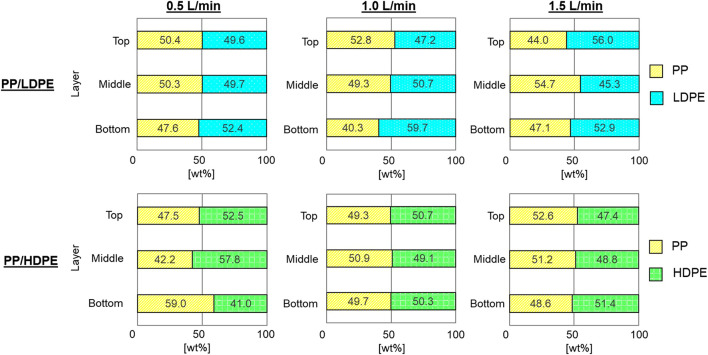
The proportion of plastics in each layer after reverse hybrid jig separation of PP/LDPE and PP/HDPE with continuous bubble generation at different air flow rate of 0.5, 1.0, and 1.5 L/min in water (i.e., without wetting agent).

**FIGURE 6:**
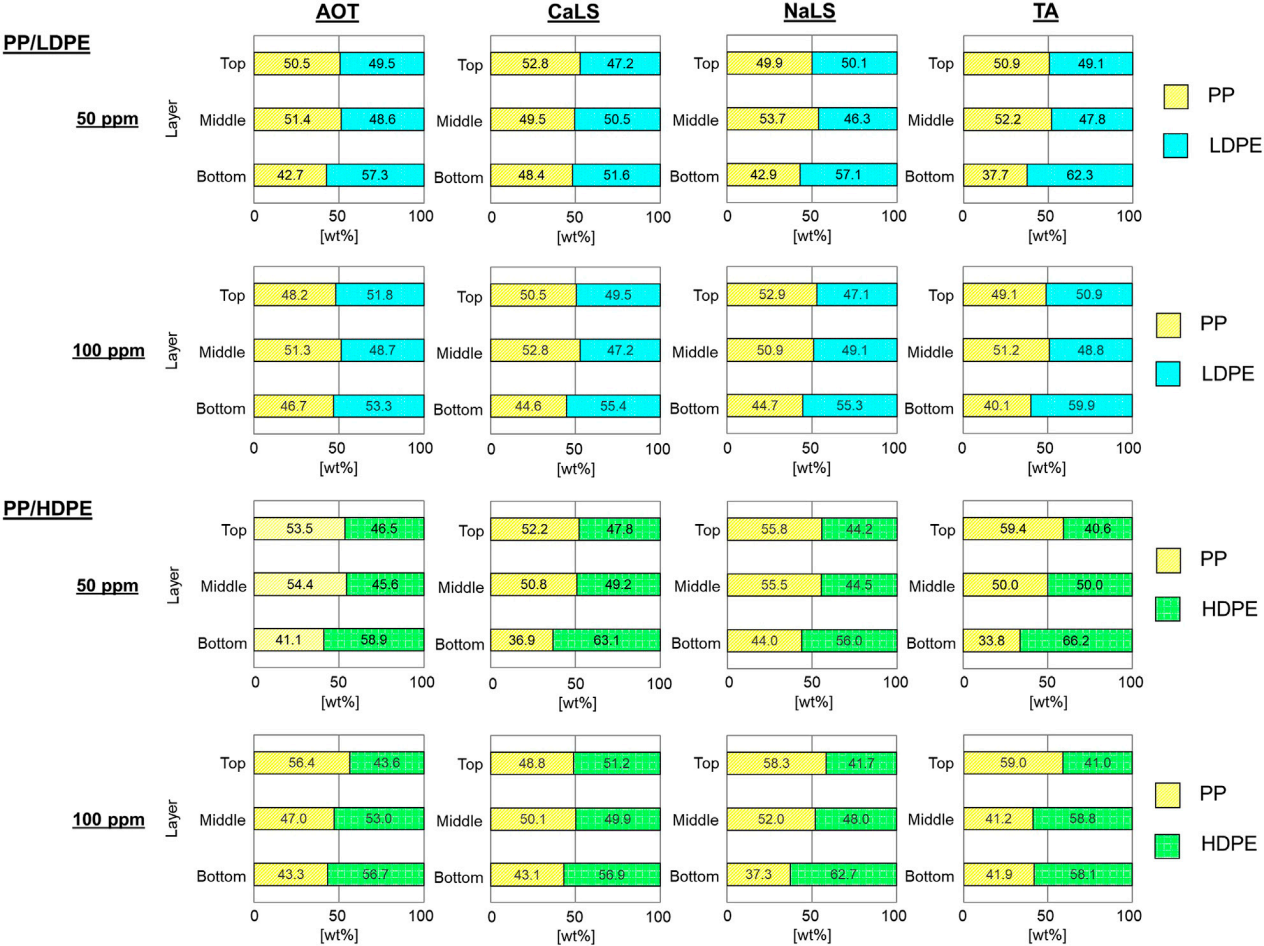
The proportion of plastics in each layer after reverse hybrid jig separation of PP/LDPE and PP/HDPE with continuous bubble generation at air flow rate of 1.0 L/min in the solution with AOT, CaLS, NaLS, and TA at concentration of 50 and 100 ppm.

The results of reverse hybrid jig separation experiments with continuous bubble generation of PP/HDPE and PP/LDPE plastic mixtures using various air flow rates as well as different wetting agent types and concentrations are presented in Figure 5. It was found that after separation, each layers contained similar amounts of PP and PE (i.e., LDPE or HDPE), indicating that separation did not occur. Visual observations suggest that separation was disturbed by the rising motion of air bubbles, which influence the fluidization behavior of the particle bed during water pulsation. However, there were slight improvements in PP/HDPE separation when wetting agents, particularly in TA, were added (Figure 6). These results confirmed that wetting agents could change the surface wettability of HDPE particles and improve the efficiency of reverse hybrid jig separation.

**FIGURE 7:**
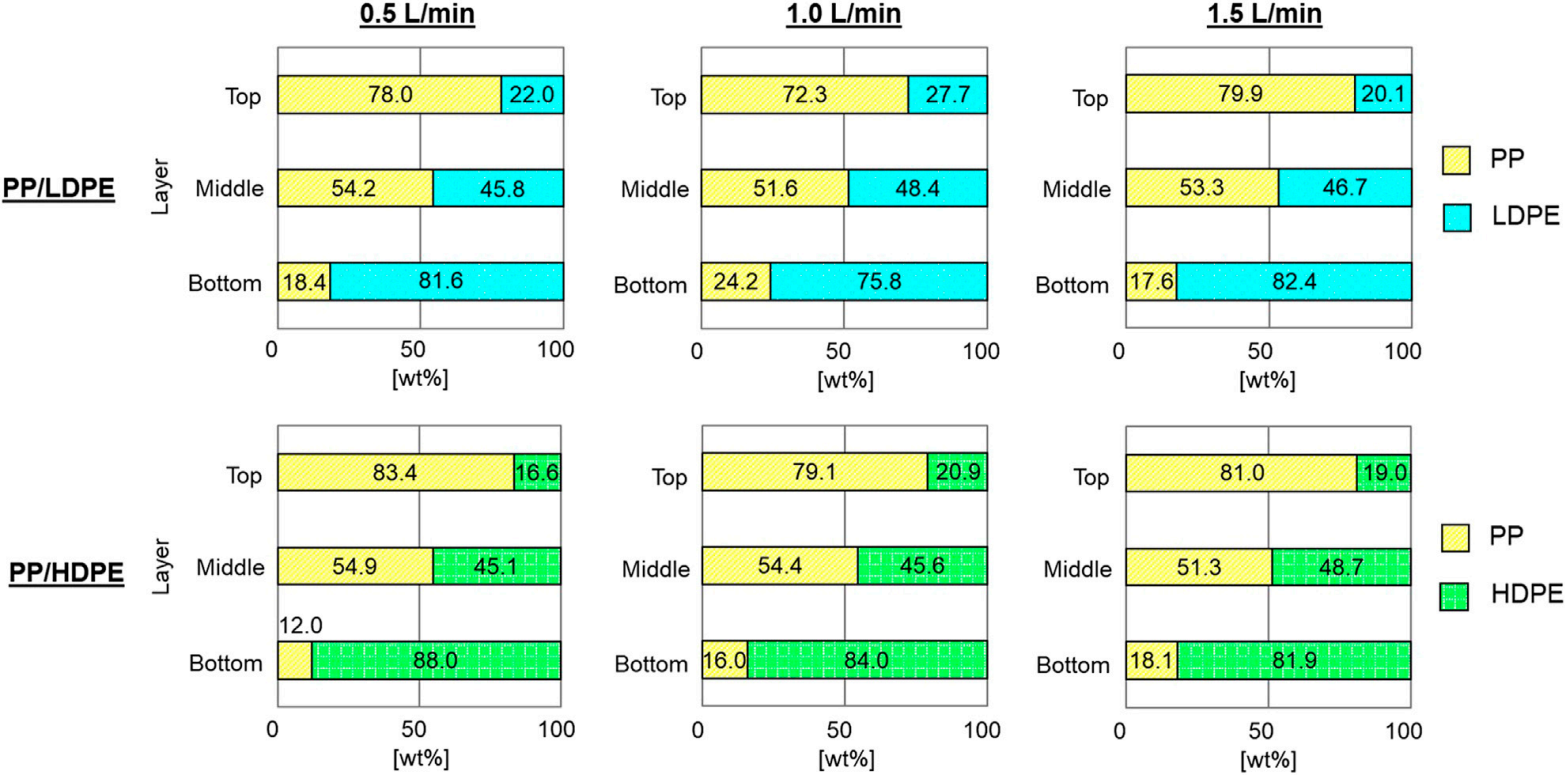
The proportion of plastics in each layer after reverse hybrid jig separation of PP/LDPE and PP/HDPE without bubble generation during pulsation at different air flow rates of 0.5, 1.0, and 1.5 L/min in water (i.e., without wetting agent).

**FIGURE 8:**
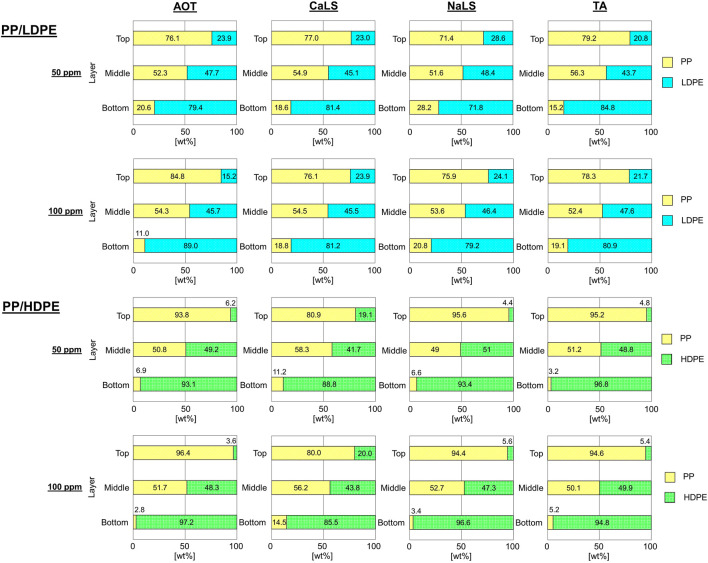
The proportion of plastics in each layer after reverse hybrid jig separation of PP/LDPE and PP/HDPE without bubble generation during pulsation at air flow rate of 1.0 L/min with AOT, CaLS, NaLS, and TA at concentrations of 50 and 100 ppm.

In contrast, the separation could occur for reverse hybrid jig separation experiments without bubble generation during pulsation (Figure 7). Compared with that of continuous bubble generation, the particle layer expanded more freely when bubbles were not introduced during pulsation. The particles with attached bubbles also stratified based on their *SG*_*A*_ without excess bubbles. Moreover, separation was possible even when *CC*_*RA*_ are less than 1.7 (Table 1). This improvement to the *CC* guide of Gupta and Yan (2016) could be attributed to the high precision control of water pulsation in advanced jig (Phengsaart et al., 2021). It was found that PP was concentrated in the top layers while PE (i.e., LDPE or HDPE) was concentrated in bottom layers. The results confirmed that separation of PP and HDPE was more effective than that of PP and LDPE in all air flow rates and was consistent with the results discussed previously (Figure 3 and Table 1). While air flow rate did not have significant effect on the separation. These indicate that *SG*_*P*_ was important for the separation. However, to prove that *SG*_*A*_ has more influence on gravity-wettability hybrid separation, more reverse hybrid jig separation experiments without bubble generation during pulsation were done with surface modification using wetting agents at air flow rate of 1.0 L/min (Figure 8 and Table 2). Similarly, PP and PE (i.e., LDPE or HDPE) were concentrated in the top and bottom layers, respectively. The results showed that the separation of PP and HDPE using AOT, NaLS, and TA improved but not for CaLS. While for PP/LDPE mixtures, the separation could not be improved. These reverse hybrid jig separation results were in line with the results of *SG*_*A*_ obtained from attached-bubbles volume measurements, indicating that separation efficiency has a stronger relationship with *SG*_*A*_ than *SG*_*P*_. Also, the *CC*_*RA*_ could be used to estimate the efficiency of reverse jig separation.

### 4.3 Relationship of sharpness index of reverse hybrid jig separation without bubble generation during pulsation and reverse apparent concentration criterion.

To determine the separation efficiency, purity distribution curves (Supplementary Figure S3, S4) were plotted from the results of Figure 7 and Figure 8, respectively. The horizontal axis (*X*) refers to the height (distance between the center position of each product layer and the lowest point of particle bed) and the vertical axis is the purity of PP while the horizontal axis (*Y*) refers to the purity of lighter plastic (i.e., PP) of each layer products.

From these purity distribution curves, the sharpness index (*SI*) could be calculated by Eq. 23 (Ito et al., 2020).

$$SI=\frac{X_{84.13}-X_{50}}{X_{50}}$$ (23)

where *X*_*84.13*_ and *X*_*50*_ are the heights when purities of PP are 84.13 and 50%, respectively.

The value of *CC*_*RA*_ and *SI* under various conditions of air flow rate and surface modifications using wetting agents are shown in Table 1 and Table 2. It was found that the *SIs* were low (high separation efficiency) when *CC*_*RA*_ were high.

**FIGURE 9:**
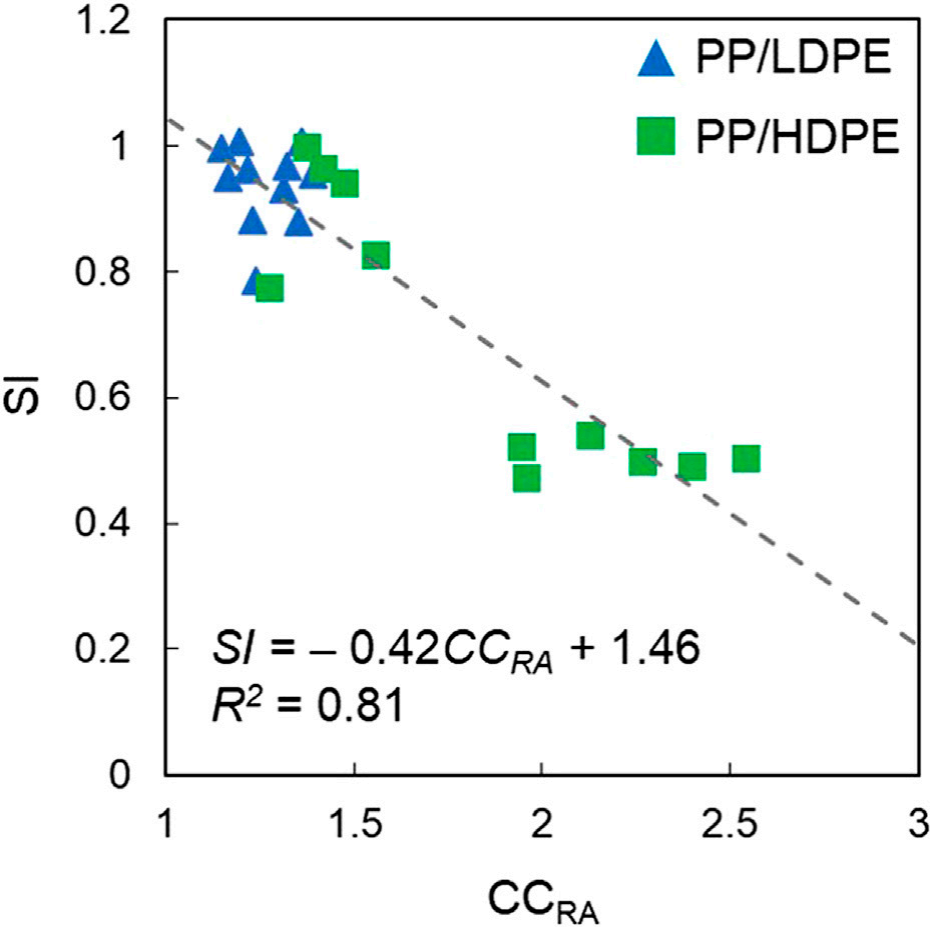
Relationship between sharpness index (*SI*) and reverse apparent concentration criterion (*CC*_*RA*_).

To understand the relationship of *CC*_*RA*_ and *SI* more clearly, the data in Table 1 and Table 2 were plotted into Figure 9. An empirical linear equation (Eq. 24) as a function of *CC*_*RA*_ and *SI* was obtained using least-squares method with a coefficient of determination (*R* ^*2*^) of 0.81.

$$SI=-0.42{CC}_{RA}+1.46$$ (24)

This relationship showed that the higher the values of *CC*_*RA*_, the lower are the values of *SI*, which means that higher separation efficiency and *CC*_*RA*_ as well as attached-bubble volume measurement could be used to estimate the reverse jig separation efficiency when air bubbles are not introduced during water pulsation.

## 5 Conclusions

In this study, a new index called the reverse apparent concentration criterion (*CC*_*RA*_) is proposed to estimate the efficiency of reverse jig separation at various air flow rates and surface modifications using wetting agents. The findings of this study are summarized as follows:

• Higher air flow rate deceased the *SG*_A_ of plastics because more air bubbles were generated and attached on plastic surfaces.
• Reverse hybrid jig separation with continuous bubble generation could not be used to separate mixed plastics because the rising motion of air bubbles disturbed particle fluidization. By stopping bubble generation during pulsation, separation occurred because the particle bed could expand more freely.
• Except for CaLS, AOT, NaLS, and TA could selectively modify the surface of HDPE from hydrophobic to become more hydrophilic that improved the separation of PP/HDPE plastic mixtures.
• *CC*_RA_, a modified *CC* for reverse hybrid jig, obtained using *SG*_A_ and *V*_0_^*^ from the attached-bubble volume measurement could be used to estimate the efficiency of reverse hybrid jig separation.

## Data Availability

The original contributions presented in the study are included in the article/Supplementary Material, further inquiries can be directed to the corresponding author.
