# Supplementary material for: Reverse hybrid jig separation efficiency estimation of floating plastics using apparent specific gravity and concentration criterion
Source: Front Chem. 2022 Sep 28;10:1014441. doi: 10.3389/fchem.2022.1014441 (PMC9554246; doi:10.3389/fchem.2022.1014441)
Supplement: Supplementary file 1 [file DataSheet1.docx]

**SUPPLEMENTARY MATERIALS**


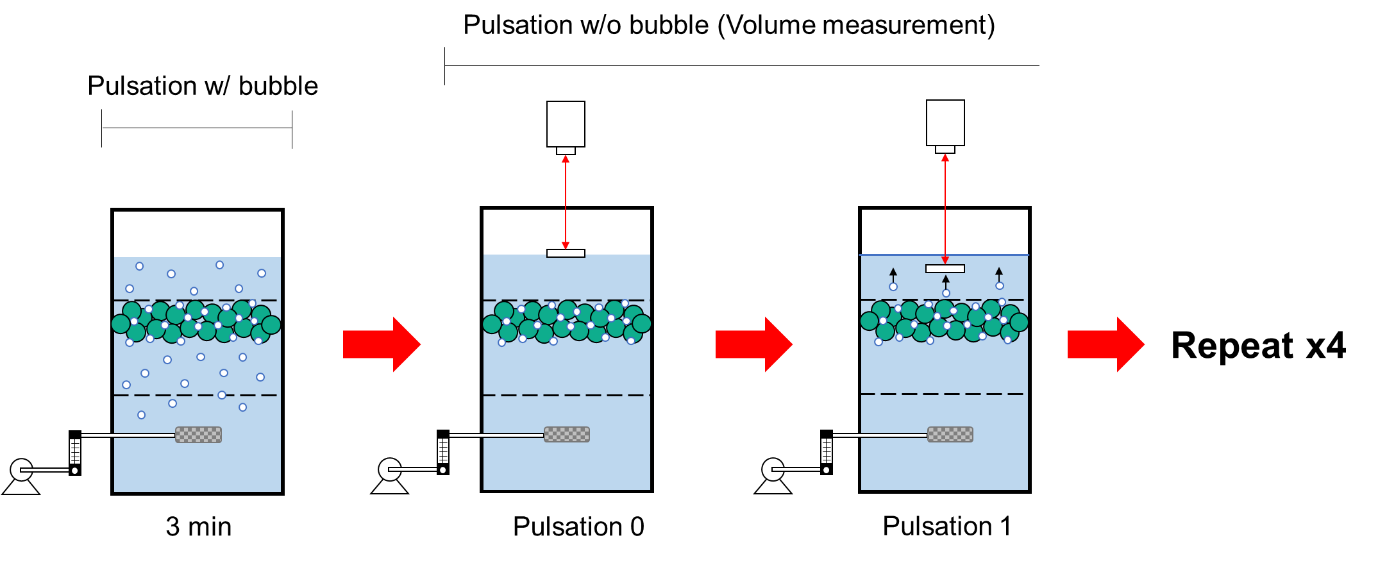


**FIGURE S1. A schematic diagram of attached-bubble volume measurement procedure.** Firstly, air bubbles were generated to attach on plastic samples under water pulsation for 3 min. Then, aeration was stopped, and level of water surface was measured by laser-based level sensor using a floating reflector on top of the water surface. Next, 1 cycle of water pulsation was created to remove trapped bubbles and level of water surface was measured again to remove bubbles trapped in the void between plastic particles. This step was repeated for 4 times.


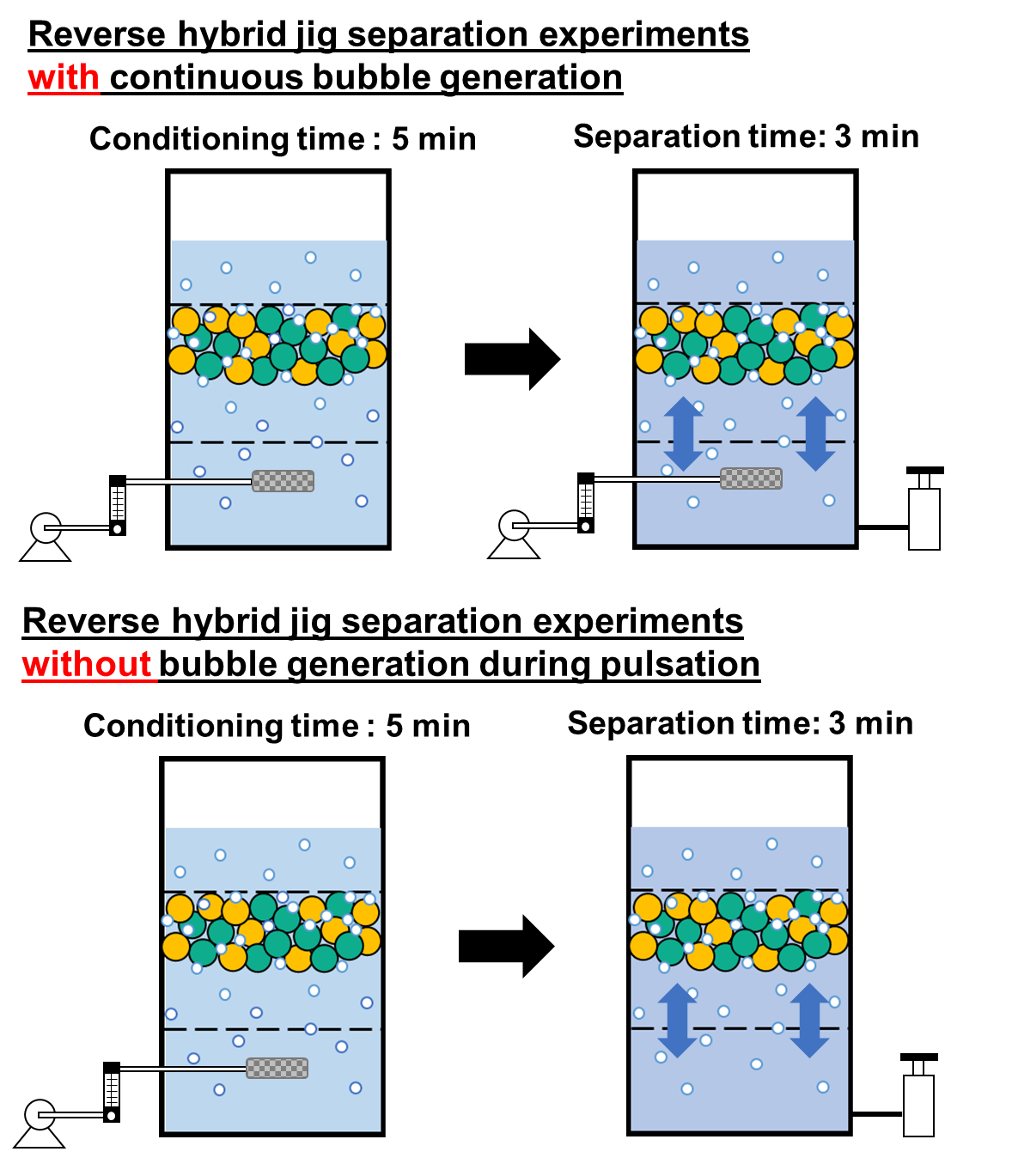


**FIGURE S2.** **Reverse hybrid jig separation experiments with continuous bubble generation and without bubble generation during pulsation.** For the first separation method, air bubbles were introduced for 5 min as a kind of “conditioning” process. This was followed by water pulsation for 3 min with continuous bubble generation bubble. While for the second separation method, air bubbles were also introduced for 5 min, but after conditioning, air introduction was stopped and water pulsation was introduced for 3 min.


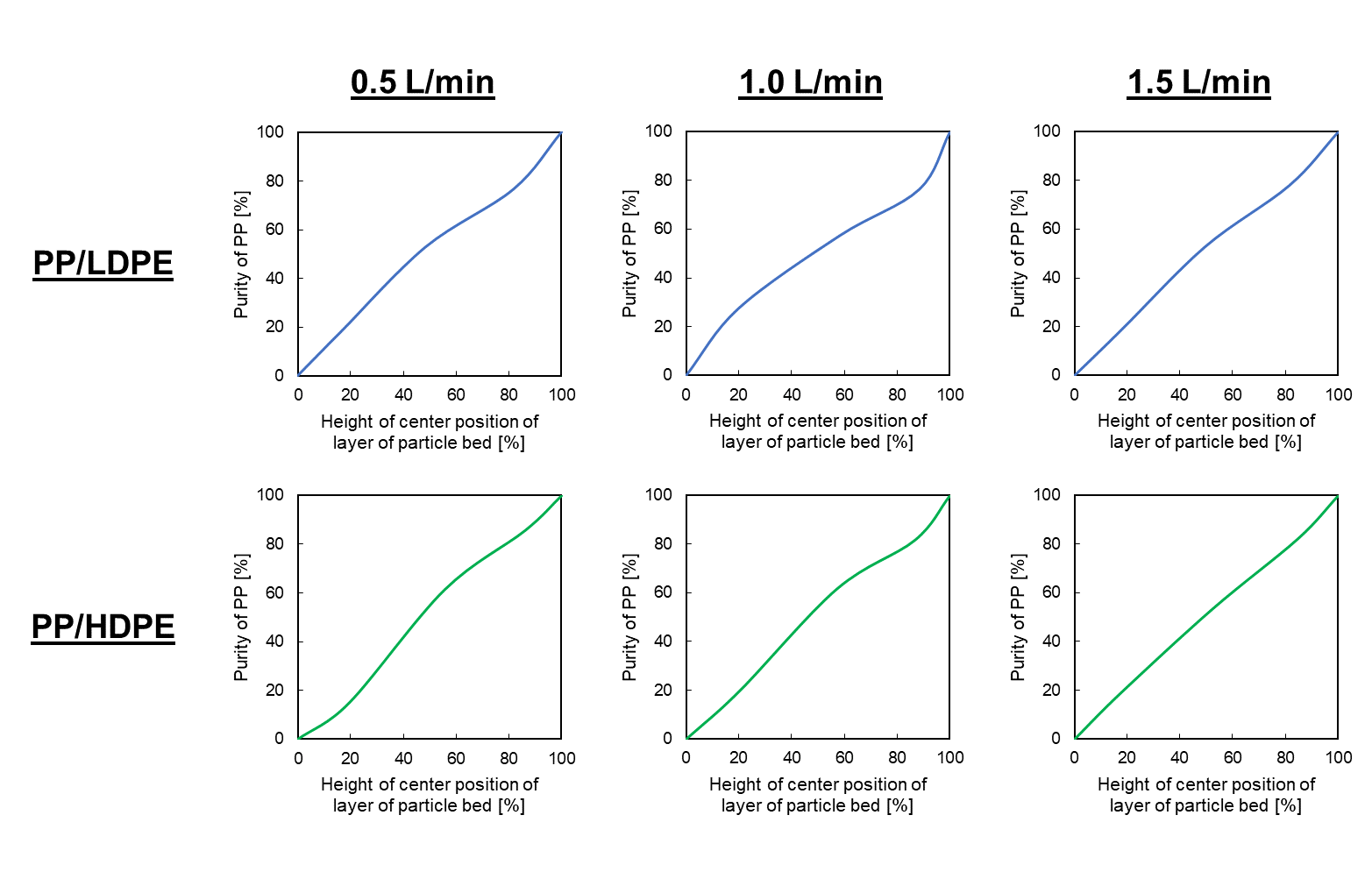


**FIGURE S3. Purity distribution curves after reverse hybrid jig separation of PP/LDPE and PP/HDPE** **without bubble generation during pulsation at different air flow rate of 0.5, 1.0, and 1.5 L/min in water (i.e., without wetting agent).** The horizontal axis (*X*) refers to the height (distance between the center position of each product layer and the lowest point of particle bed) and the vertical axis is the purity of PP while the horizontal axis (*Y*) refers to the purity of lighter plastic (i.e., PP) of each layer products.

**
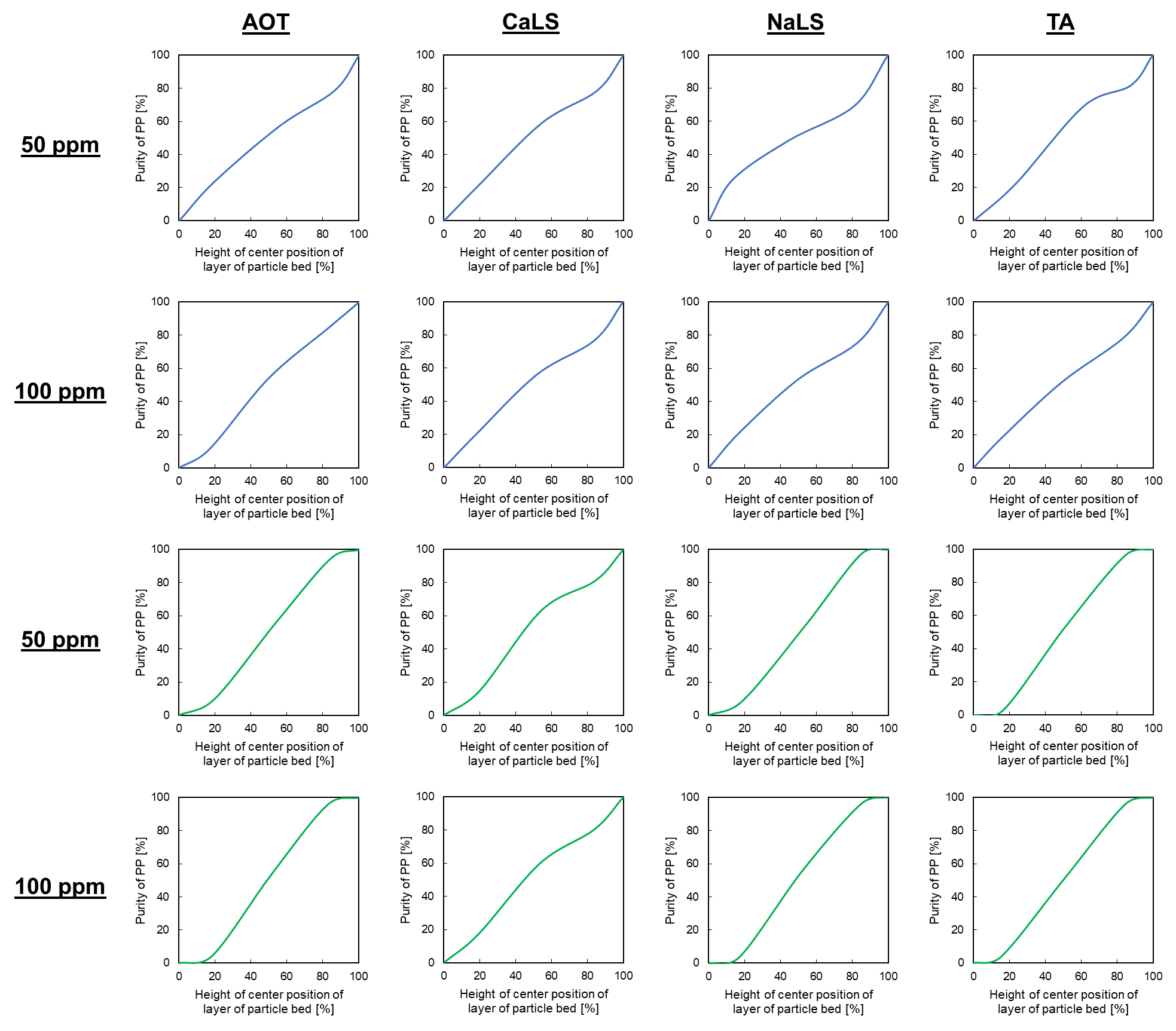
**

**FIGURE S4. Purity distribution curves after reverse hybrid jig separation of PP/LDPE** **and PP/HDPE without bubble generation during pulsation at air flow rate of 1.0 L/min in the solution with AOT, CaLS, NaLS, and TA at concentration of 50 and 100 ppm.** The horizontal axis (*X*) refers to the height (distance between the center position of each product layer and the lowest point of particle bed) and the vertical axis is the purity of PP while the horizontal axis (*Y*) refers to the purity of lighter plastic (i.e., PP) of each layer products.
